# Gingival fibroblasts prevent BMP‐mediated osteoblastic differentiation

**DOI:** 10.1111/jre.12631

**Published:** 2018-12-03

**Authors:** Mandeep S. Ghuman, Maher Al‐Masri, Guilherme Xavier, Martyn T. Cobourne, Ian J. McKay, Francis J. Hughes

**Affiliations:** ^1^ Division of Tissue Engineering and Biophotonics Dental Institute King's College London London UK; ^2^ BPP Dental Institute BPP University Birmingham UK; ^3^ Centre for Craniofacial and Regenerative Biology Dental Institute King's College London Guy's Hospital London UK; ^4^ Department of Adult Oral Health Institute of Dentistry, Barts and The London School of Medicine and Dentistry Queen Mary University of London London UK

**Keywords:** bone, bone morphogenetic proteins, conditioned, culture media, paracrine communication

## Abstract

**Objectives:**

The inhibitory action of the superficial gingival connective tissues may limit the regenerative potential of alveolar bone in periodontal therapy or dental implant applications. The aims of this study were to investigate the hypothesis that gingival fibroblasts (GF) can inhibit bone morphogenetic protein (BMP)‐induced osteoblastic differentiation, to determine their expression of BMP inhibitors, and finally to determine whether reduction of these inhibitors can relieve suppression of osteoblastic differentiation.

**Methods:**

Gingival fibroblasts were co‐cultured either directly or indirectly with calvarial osteoblasts to assess alkaline phosphatase inhibitory activity, a marker of osteoblastic differentiation. To test total BMP‐inhibitory activity of rat GF, conditioned media (GFCM) were collected from cultures. ROS 17/2.8 osteoblastic cells were stimulated with BMP2, together with GFCM. Inhibitor expression was tested using RT‐qPCR, Western blotting and in situ hybridization. Removal of inhibitors was carried out using immunoprecipitation beads.

**Results:**

Co‐culture experiments showed GF‐secreted factors that inhibit BMP‐stimulated ALP activity. 10 ng/ml BMP2 increased alkaline phosphatase expression in ROS cells by 41%. GFCM blocked BMP activity which was equivalent to the activity of 100 ng/ml Noggin, a well‐described BMP inhibitor. Cultured gingival fibroblasts constitutively expressed BMP antagonist genes from the same subfamily, *Grem1*,* Grem2* and *Nbl1* and the Wnt inhibitor *Sfrp1*. Gremlin1 (6.7 × reference gene expression) had highest levels of basal expression. ISH analysis showed Gremlin1 expression was restricted to the inner half of the gingival lamina propria and the PDL. Removal of Gremlin1 protein from GFCM eliminated the inhibitory effect of GFCM on ALP activity in ROS cells. Subsequent addition of recombinant Gremlin1 restored the inhibitory activity.

**Conclusions:**

Factors secreted by gingival fibroblasts inhibit BMP‐induced bone formation and a range of BMP inhibitors are constitutively expressed in gingival connective tissues. These inhibitors, particularly Gremlin1, may limit coronal alveolar bone regenerative potential during oral and periodontal surgery.

## 1 INTRODUCTION

New bone formation in periodontal regenerative treatments is generally restricted to infrabony sites. With a horizontal pattern of bone loss, regeneration is typically limited by encroachment of the overlying gingival connective tissues. Undesirable invasion by soft connective tissue negatively influences both the extent and nature of the bone formed;^1^ whilst elsewhere in the body clinical, developmental and experimental observations suggest that bone formation can be inhibited by non‐ossifying connective tissues.^2^ The biological basis for this effect may be due to competition for the available space adjacent to bone; however, an alternative hypothesis is that fibroblasts constitutively exert inhibitory activity that limits bone growth.

Bone morphogenetic proteins (BMPs) are critical for osteogenesis and are unique in their ability to induce bone formation de novo in ectopic sites^3^ by induction of osteoblastic commitment of multipotent mesenchymal stem cells.^4^, ^5^ In rodent and feline mandibular fenestration defect models, BMP2 enhances healing and regeneration of bone tissue but does not lead to lasting ankylosis.^6^, ^7^ Interestingly, doses well in excess of the physiological range are required to induce significant bone formation. BMP activity is tightly regulated at both the extra and intracellular levels, including by secretion of BMP antagonists such as Gremlin1 and Noggin.^8^ Therefore, it is suggested that osteogenic inhibitory influences from connective tissue may involve these inhibitors.

The aim of this study was to test the hypothesis that bone formation is inhibited by the production of inhibitory factors within the gingival connective tissues. Our specific objectives were to test whether gingival cells inhibited osteoblast differentiation, to characterize the expression of bone inhibitory factors by the gingiva and to identify specific inhibitory factors which may be responsible for this activity.

## 2 MATERIALS AND METHODS

### 2.1 Cell cultures

Primary fibroblasts were isolated from gingivae of 7‐8 week post‐natal male Wistar rats (180‐200 g) (Charles Rivers, Margate, UK). Animal tissues were derived in accordance with UK Home Office approved animal use protocols. Briefly, immediately after sacrifice, samples were harvested using scalpels and minced into small fragments before plating onto culture dishes to allow cell outgrowth. Primary osteoblasts were isolated from calvariae of 1‐3 day post‐natal Wistar rats by sequential collagenase digestion as previously described.^9^ Primary rat femoral bone marrow stromal cells (BMSCs) were isolated from 5‐6 week post‐natal male Wistar rats (100‐120 g) as previously described.^10^ MC3T3‐E1 mouse pre‐osteoblastic (MC3T3) cells were purchased from ATCC (catalogue number CRL‐2593). All cell cultures were expanded using α‐MEM (Gibco, Paisley, UK) supplemented with 10% newborn calf serum (NCS) (First Link, Birmingham, UK), 50 U/ml penicillin, 50 ug/ml streptomycin, 0.3 μg/ml Fungizone (both Gibco, Paisley, UK) termed standard medium. The rat osteosarcoma cell line ROS 17/2.8^11^ was cultured with standard medium. Details of fibroblast‐osteoblast collagen gel and transwell co‐culture methods can be found in the Supporting information Appendix S1.

### 2.2 Media conditioning

Nearly, confluent primary cell cultures were washed three times with PBS prior to addition of serum‐free standard media. Conditioned media (CM) were collected after 24 hours, passed through 0.22 μm filters (Nunc, Loughborough, UK) to remove any cellular debris and stored at −20°C until further use. Prior to use in experiments, 10% NBCS was added to CM. Details of methods to characterize conditioned media can be found in the Supporting Material file.

### 2.3 Osteoblast proliferation and differentiation assays

5 × 10^3^ ROS17/2.8 or primary calvarial cells were seeded in 96‐well plates for 24 hours in standard media. Cell cultures were treated with conditioned media and recombinant mouse (rm)BMP2, rmGremlin1 or rmNoggin (all from R&D Systems, Abingdon, UK). 72 hours following treatment proliferation was assessed using MTS assay according to the manufacturer's instructions (CellTiter 96 AQ_ueous_ One Solution Cell Proliferation assay; Promega, Southampton, UK) followed immediately by an assay of alkaline phosphatase (ALP) activity to assess osteoblastic differentiation. ALP activity was assayed as described previously^12^ (all reagents from Sigma‐Aldrich, Gillingham, UK).

### 2.4 Migration assays

Migration of calvarial cells was tested using a 48 well chemotaxis chamber according to our previously described methods.^13^ Details are shown in Supporting information Appendix S1. Migration of cells towards recombinant rmBMP2 or rmPDGFbb (R&D Systems) over 4 hours was tested.

### 2.5 RT‐qPCR analysis

Total RNA was extracted from cultured cells and expression of secreted BMP and Wnt inhibitors was determined by RT‐qPCR with TaqMan Gene expression assays (Applied Biosystems, Warrington, UK) as described in detail in the Supporting information Appendix S1.

### 2.6 In situ hybridization

Mouse *Grem1, Grem2, Nbl1 and Nog* riboprobes were generated from clones kindly supplied by Professor Richard Harland, Department of Molecular & Cell Biology, University of California, Berkeley. Plasmid and probe generation information is summarized in Tables 1 in the Supporting information Appendix S1. Plasmid DNA was linearized and antisense ^35^S‐UTP radio‐labelled riboprobes generated.

Eight‐week‐old wild‐type male C57BL/6 mice were killed by cervical dislocation. Mandibles and maxillae were isolated and fixed in 4% (w/v) paraformaldehyde overnight, washed with PBS, then decalcified with Morse's solution.^14^ End point of decalcification was determined chemically by testing residual calcium. Specimens were then dehydrated through a graded ethanol series. Following this, specimens were then embedded in paraffin wax and sectioned at 7 μm, prior to section in situ hybridization.

Section radioactive in situ hybridization* *was carried out as previously described.^15^ Light‐ and dark‐field images of sections were photographed using a Zeiss Axioscop microscope and merged in Adobe Photoshop CS.

### 2.7 Immunoprecipitation

The Dynabeads Protein G IP kit (Invitrogen, Paisley, UK) was used for immunoprecipitation of Gremlin1 from conditioned media according to the manufacturer's instructions. Details are shown in the Supporting information Appendix S1.

### 2.8 Western blotting analysis

The presence of Gremlin1 protein was tested in conditioned media and in eluted proteins from IP beads, by Western Blot. Details are shown in the Supporting information Appendix S1.

### 2.9 Statistical analysis

Data are expressed as means ± SD. To allow meaningful comparison between different experiments, the values were normalized to the control (background) levels of ALP adjusted for cell number. One‐way ANOVA was used to determine whether differences in alkaline phosphatase activity and expression of inhibitors in the different cell type were statistically significant from controls. All ANOVA analyses were examined post hoc by Bonferroni's post‐test. Statistical analysis was carried out using Prism v5 software (GraphPad Software Inc., California). The statistical significance was set at *P* < 0.05.

## 3 RESULTS

### 3.1 Gingival fibroblasts inhibit osteoblastic differentiation

**Figure 1:**
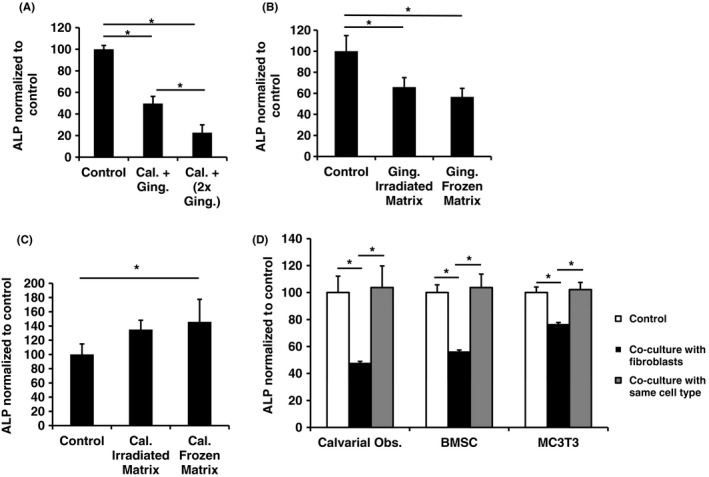
A, Effect of gingival fibroblasts on ALP activity in collagen matrix culture systems. Gingival fibroblasts were seeded with calvarial osteoblasts into collagen matrices. Control: ALP level of 5 × 10^5^ osteoblasts grown in matrices containing 1 × 10^6^ osteoblasts. Cal. + Ging.: ALP level of 5 × 10^5^ osteoblasts grown in matrices with 5 × 10^5^ gingival fibroblasts. Cal. + (2 × Ging.): ALP level of 5 × 10^5^ osteoblasts grown in matrices with 1 × 10^6^ gingival fibroblasts. B and C, Effect of gingival fibroblast (B) and calvarial osteoblast (C) matrices on calvarial ALP activity. Control: ALP level of cultured calvarial osteoblasts in no matrix. Irradiated Matrix: ALP level osteoblasts grown on gingival or osteoblastic matrices obtained by irradiation. Frozen Matrix: ALP level of calvarial osteoblasts grown on gingival or osteoblastic matrices obtained by freeze/thaw cycles. D, Inhibition of total ALP activity of cells of the osteoblastic lineage co‐cultured in the presence of gingival fibroblasts. Gingival fibroblasts were seeded onto cell culture membrane inserts and placed into wells 24 hours after seeding of either primary rat calvarial osteoblasts, primary rat bone marrow stromal cells (BMSC) and the murine osteoblastic line MC3T3‐E1. ALP levels measured 72 hours after adding fibroblasts. Control: Osteoblast lineage cells cultured in wells without cells on inserts. Co‐culture with same cell type: Osteoblast lineage cells grown on both surfaces. All values normalized to control. ALP activity adjusted for cell number in all experiments. Minimum of three replicates per experiment. Data shown as mean ± SD. Representative data from three independent experiments are shown. Significant differences shown as * (*P* < 0.05). Significance tested using one‐way ANOVA with Bonferroni post‐tests

Gingival fibroblasts inhibited differentiation of primary calvarial osteoblasts when cultured with an equal number of gingival fibroblasts seeded simultaneously in collagen matrices (Figure 1A). Furthermore, the inhibition was further enhanced to a significant level when gingival fibroblast number was doubled. Gingival fibroblasts similarly inhibited differentiation when seeded 24 hours after calvarial osteoblasts in collagen matrices, implicating a paracrine mechanism due to reduced direct fibroblast to osteoblast contact. However, inhibition was not significantly further enhanced by doubling gingival fibroblast number (Supporting information Figure S1).

To test whether live fibroblasts were needed to produce the previously observed inhibition of osteoblastic differentiation, the effect of cell matrices was tested. Osteoblastic differentiation was significantly inhibited when calvarial osteoblasts were seeded onto gingival fibroblast matrices obtained following irradiating cells by UV irradiation for 18 hours (Supporting information Figure 1B). When matrices were obtained following repeated freeze‐thaw cycles, a similar level of inhibition was achieved. In contrast, irradiated calvarial osteoblast matrices did not significantly alter osteoblastic differentiation, whereas the matrix obtained after freeze‐thawing significantly enhanced differentiation (Figure 1C).

In transwell cultures, ALP levels were significantly reduced in cells tested at different stages of the osteoblast lineage—primary calvarial osteoblasts, bone marrow stromal stem cells and MC3T3 cells—when co‐cultured with fibroblasts (Figure 1D). Co‐culture with the same cells did not significantly alter osteoblastic differentiation.

### 3.2 Gingival fibroblast conditioned media inhibits osteoblastic differentiation

**Figure 2:**
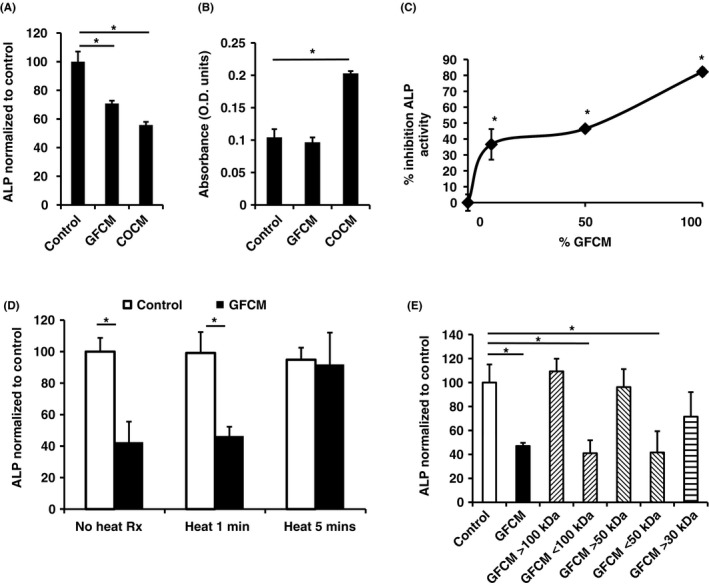
A, Inhibition of ALP activity of calvarial osteoblasts when treated with different conditioned media (CM). B, Parallel cell proliferation assay of osteoblasts treated with different CM. Control, standard media; GFCM, Gingival fibroblast CM; COCM, Calvarial osteoblast CM. C, Inhibition of ROS cell ALP activity with dilution of GFCM. GFCM diluted with standard media. D, Normalized ROS cell ALP activity treated with heat‐treated (1‐10 minutes) GFCM. Control: standard media. Heat treatment—GFCM placed in boiling water for 1, 5 or 10 minutes. E, Normalized ROS cell ALP activity of treated with molecular weight fractionated GFCM. > indicates concentrates, < filtrates. ALP activity adjusted for cell number in all experiments. Minimum of three replicates per experiment. Data shown as mean ± SD. Representative data from three independent experiments are shown. Significant differences shown as *(*P* < 0.05). Significance tested using one‐way ANOVA with Bonferroni post‐tests

Gingival fibroblast conditioned media (GFCM) significantly inhibited calvarial osteoblast differentiation (Figure 2A), whilst having no significant effect on proliferation (Figure 2B). Interestingly, calvarial osteoblast conditioned medium also lowered ALP levels (Figure 2A) which could be explained by its effect of stimulating osteoblast proliferation thus diminishing a differentiation response (Figure 2B).

GFCM were subsequently applied to the rat osteosarcoma cell line ROS 17/2.8 in order to establish a reproducible model of osteoblastic differentiation in terms of ALP levels. Dilution of GFCM resulted in a non‐linear reduction in inhibitory activity retaining approximately 40% of the effect at a 1:10 dilution (Figure 2C).

### 3.3 Further characterization of GFCM activity

Inhibitory activity of GFCM was heat‐labile, being rendered inactive after 5 minutes of boiling (Figure 2D). To further confirm whether GFCM inhibitory activity was proteinaceous in nature, trypsin was added to GFCM. Inhibitory activity was abolished by incubation with trypsin for 2 hours (after which protease inhibitor was added to inactivate residual trypsin to allow the GFCM to be subsequently assayed). Incubation of protease inhibitor alone had no effect on ROS cell ALP activity (Supporting information Figure S2). Fractionation of GFCM by molecular mass revealed inhibitory activity was maintained in fractions below 50 kDa, and activity was partially lost in fractions above 30 kDa (Figure 2E).

### 3.4 BMP2 overcomes the inhibitory activity of GFCM

**Figure 3:**
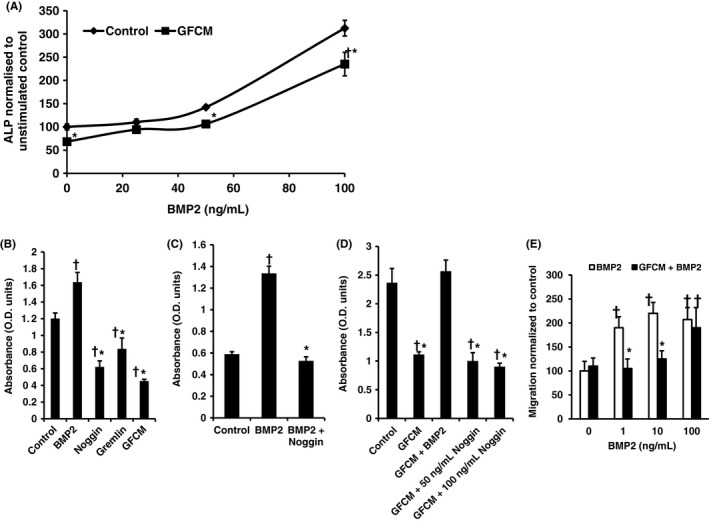
A, Normalized osteoblastic ALP activity cultured in gingival fibroblast conditioned media (GFCM) treated with BMP2. B, ROS cell ALP activity following treatment with 10 ng/ml BMP2, 100 ng/ml Noggin, 100 ng/ml Gremlin1 and GFCM. Single dose stimulations employed. C, ROS cell ALP activity stimulated by 10 ng/ml BMP2 is inhibited by 100 ng/ml noggin. Single dose stimulations employed D. 10 ng/ml BMP2 overcomes inhibitory effect on ALP activity of GFCM in ROS cells. Single dose stimulations used. Control in all experiments was standard media. ALP activity assessed after 72 hours and adjusted for cell number in all experiments. E, Inhibitory effect of GFCM on BMP2‐stimulated calvarial osteoblast chemotaxis. Five counts per well. Minimum of three replicates per experiment. Data shown as mean ± SD. Representative data from three independent experiments are shown. Significant differences shown as *P < 0.05 to BMP‐matched control, ^†^ *P* < 0.05 to baseline control. Significance tested using one‐way ANOVA with Bonferroni post‐tests

Increasing BMP2 concentrations partially reversed the inhibitory effects of GFCM on osteoblastic differentiation in both primary calvarial osteoblasts (25 ng/ml) (Figure 3A) and ROS cells (10 ng/ml) (Supporting information Figure S3) restoring ALP activity to baseline control levels. In ROS cells, the inhibitory activity of GFCM was completely reversed by addition of 25 ng/ml BMP2.

We next investigated whether this effect was similar to that caused by known BMP antagonists. 100 ng/ml Gremlin1 and 100 ng/ml Noggin inhibited osteoblastic differentiation to a similar extent as that caused by GFCM (Figure 3B). 100 ng/ml noggin completely inhibited the stimulatory effect of 10 ng/ml BMP2 in control cultures (Figure 3C), whereas addition of 10 ng/ml BMP2 completely overcame inhibition of GFCM (Figure 3D). GFCM therefore possessed BMP inhibitory activity that mimicked the action of BMP antagonists and was equivalent to approximately 100 ng/ml noggin. Furthermore, the inhibitory effect of GFCM appears to be saturated as it was not enhanced further by the addition of either 50 ng/ml or 100 ng/ml noggin (Figure 3D). GFCM also significantly inhibited calvarial osteoblast BMP‐stimulated chemotaxis at concentrations up to 10 ng/ml (Figure 3E). Again, this was to a similar extent as that caused by 50 ng/ml noggin but GFCM did not inhibit PDGF‐stimulated chemotaxis indicating selective inhibition (data not shown).

### 3.5 BMP inhibitor expression in gingival tissues

**Figure 4:**
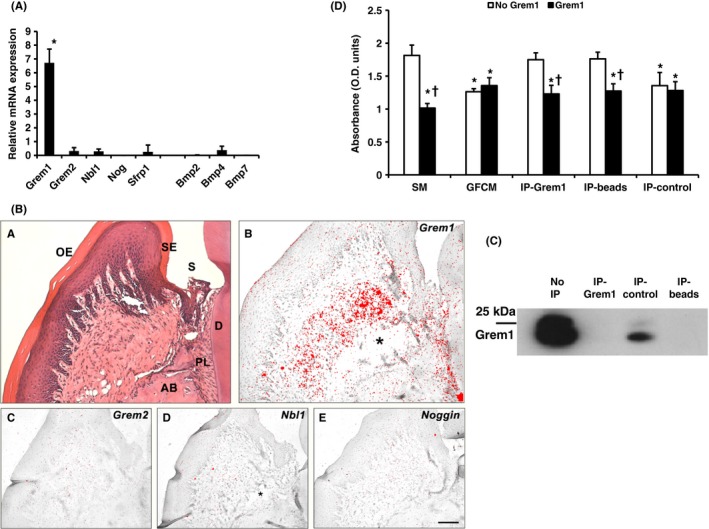
A. Relative expression of BMP and BMP inhibitor genes by cultured gingival fibroblasts Expression normalized to averaged *Eif4a2* and *Atp5b* expression. Data shown as mean ± SD (n = 5 animals). Assays carried out in triplicate. *P < 0.05—*Grem1* expression significantly higher than all others tested. Significance tested by one‐way ANOVA with Bonferroni post‐tests. B, In situ hybridization of BMP inhibitor mRNA expression in molar periodontal tissue of 8 wk post‐natal WT mice. A, H&E staining, B, *Grem1* expression restricted to the inner half of the gingival lamina propria closest to the alveolar bone and periodontal ligament. C‐E, Expression of *Grem2* (D), *Nbl1* (E) and Noggin (F) undetectable. OE, oral epithelium; SE, sulcular epithelium; S, sulcus; D, dentine; PL, periodontal ligament; AB, alveolar bone; *, processing artefact. Scale bar in *F* = 100 μm for A‐E (n = 2 mice). Results confirmed in two independent experiments. C, Western blotting of gingival fibroblast conditioned media confirming the presence of Gremlin1 and removal with immunoprecipitation beads. Lanes: No IP—Concentrated gingival fibroblast conditioned media containing proteins >5 kDa, IP‐Grem1—Concentrated GFCM treated with IP beads with Gremlin1 antibody adsorbed, IP‐control—Concentrated GFCM treated with IP beads with isotype control antibody adsorbed, IP‐beads—GFCM treated with IP beads without antibody adsorbed. 10 μg total protein/well loaded. D, Inhibition of ROS cell ALP activity of Gremlin1‐depleted gingival fibroblast conditioned medium is rescued with addition of Gremlin1. GFCM that underwent IP were analysed in the ROS cell ALP activity assay. SM—standard media. GFCM—concentrated GFCM. IP‐Grem1—Concentrated GFCM treated with IP beads with Gremlin1 antibody adsorbed. IP‐beads—GFCM treated with IP beads without antibody adsorbed. IP‐control—Concentrated GFCM treated with IP beads with isotype control antibody adsorbed, Grem1—100 ng/ml rmGremlin1 added immediately prior to ROS assay. No Grem1—no Gremlin1 added. Results shown as mean ± SD. *—significantly different from standard media with no Gremlin1, †—significantly different from matched sample without Gremlin1 added. *P* < 0.05. Each treatment tested using five replicates from 1 cell line. Representative results from experiments testing three independent primary cell lines. Significance tested using one‐way ANOVA with Bonferroni post‐tests

Cultured gingival fibroblasts constitutively expressed genes of a number of BMP antagonists from the same DAN subfamily, *Grem1*,* Grem2* and *Nbl1* and the Wnt inhibitor *Sfrp1* (Figure 4A). *Grem1* was the most expressed inhibitor (6.5× reference gene level) at approximately 12 times the level of the next most expressed *Grem2*. Interestingly, Noggin was not expressed. Of these genes, only *Grem2* expression was regulated by BMP2, with expression significantly upregulated twofold 2 hours post‐stimulation with 100 ng/ml BMP2 before returning to baseline levels by 24 hours (data not shown).

In situ hybridization of mouse periodontal tissue revealed constitutive expression of *Grem1* restricted to the inner half of the gingival connective tissue and periodontal ligament. However, expression of *Grem2*,* Nbl1*,* Noggin* was undetectable (Figure 4B).

### 3.6 Gremlin1 contributes to the inhibitory activity of GFCM

Owing to the high expression in gingival fibroblasts, we investigated the effect of depleting Gremlin1 from GFCM on osteoblastic differentiation by immunoprecipitation (IP). Loss of inhibitory activity was subsequently assessed using the ROS cell ALP activity assay.

Gremlin1 was detectable in untreated GFCM following concentration using a 5 kDa filter (Figure 4C). Two merged bands at 22 kDa and 25 kDa were seen, mostly likely representing non‐glycosylated and glycosylated forms of Gremlin1, respectively. Gremlin1 was depleted using immunoprecipitation beads with or without anti‐Gremlin1 antibody adsorbed, indicating non‐specific removal. Adsorption of an irrelevant isotype control antibody for the neuron‐specific protein, choline acetyltransferase (ChAT) visibly removed less Gremlin1 than beads either with or without antibody to Gremlin1 bound.

IP‐depleted GFCM resulted in total loss of inhibitory activity in the ROS cell ALP activity assay (Figure 4D). In contrast, GFCM treated using beads with ChAT antibody retained an inhibitory effect on ALP activity. Inhibition of ALP activity was completely recovered by adding 100 ng/ml Gremlin1 to both GFCM treated with Gremlin1 antibody (32% reduction in ALP activity compared to the control) and with plain beads (30% reduction in ALP activity), confirming the results of the previous tests.

## 4 DISCUSSION

The results of this study show that gingival fibroblasts secrete BMP antagonists, most abundantly Gremlin1, leading to in vitro reduction of ALP activity, a marker of osteoblastic differentiation in both primary calvarial osteoblasts and a rat osteosarcoma cell line. Furthermore, depletion of this BMP antagonist resulted in a recovery of ALP activity.

To date, biological methods of enhancing bone regeneration in clinical use have centred on the promotion of osteoinduction. In vitro, BMPs are potent differentiation factors, inducing the differentiation of multipotential mesenchymal cells into osteochondrogenic lineage cells and osteoblast precursor cells.^5^, ^16^, ^17^ BMPs are expressed during alveolar bone regeneration.^18^, ^19^ Indeed, the addition of BMP2 is also able to significantly improve the results of guided tissue regeneration in human patients,^20^ but require large supraphysiological doses. Therefore, targeting BMP inhibitors may provide a novel means of improving bone regeneration and achieving other relevant osteogenic needs.

Studies of the interaction of tissues during embryogenesis suggest that mesenchymal tissues can define the boundary of bone formation by the expression of soluble molecules such as the BMP inhibitor noggin.^2^ New bone formation readily occurs in suitable environmental niches such as at a properly reduced fracture site or indeed within alveolar bone following implant placement. However, the extent of new bone formation is generally limited by the different environment surrounding the site of bone formation.

The inhibition of osteoblastic differentiation caused by gingival fibroblasts following sequential seeding in collagen gels was of a similar extent to that caused by simultaneously seeding (Figure 1 and Supporting information Figure S1) indicating that a paracrine mechanism was involved. Enhancement of inhibition caused by doubling the number of fibroblasts seeded in both cases provides evidence for a dose‐dependent nature. The inhibition caused by matrices of devitalized gingival fibroblasts demonstrated signals can persist which remain inhibitory to calvarial osteoblast ALP production. The lack of inhibition caused by calvarial matrices demonstrated the selective nature of the inhibition.

Paracrine‐mediated inhibition of osteoblastic differentiation at multiple stages of the osteoblastic lineage was shown in primary BMSCs, MC3T3, and calvarial osteoblasts when co‐cultured with gingival fibroblasts. Although calvarial osteoblasts were inhibited to the greatest degree. Collectively, the results verify gingival fibroblasts influence the differentiation activity of osteoblasts independent of physical contact.

A reproducible model of the system using ROS cells that showed a good response in terms of the inhibition of ALP synthesis was established and was also able to sensitively respond to BMP2 whilst demonstrating the antagonistic activity of BMP inhibitors. Furthermore, the lack of inhibition on PDGF‐mediated osteoblast chemotaxis by GFCM, provides further evidence of the selectivity.

The maintenance of inhibition of ROS cell ALP activity at low concentrations of conditioned medium implied complex reaction kinetics. Application of conditioned media caused no observable change to cell morphology and nutrient depletion was unlikely to be a factor due to the short period of generation. The data suggest‐high affinity binding where a low concentration of a factor is required to maintain a significant inhibition. Although exogenously applied BMP2 has been shown to regulate Gremlin1 expression in developmental models, it had no effect on expression in gingival fibroblasts, suggesting it may be regulated by an alternative BMP or different ligand class. Interestingly, Gremlin1 has also been postulated to have a BMP‐independent role in differentially modulating angiogenesis dependent upon its dimeric state via binding of vascular endothelial growth factor (VEGF) receptor 2 (VEGFR2)^21^ and is associated in renal development and upregulated in fibrotic conditions of the kidney, lung^22^, ^23^ and rheumatoid arthritis.^24^

ISH analysis showed Gremlin1 expression was found to be restricted to the inner gingival lamina propria closest to the alveolar bone and periodontal ligament suggesting the presence of regionally specific subpopulations of fibroblasts, as with the dermis.^25^ Fibroblasts that populate the superficial papillary dermis have been shown to be morphologically and physiologically distinct from fibroblasts derived from the deeper reticular dermis.^26^ Irwin et al^27^ compared human gingival cells derived from the two layers and showed that papillary fibroblasts proliferated faster and were smaller and more spindly. Groeneveld et al^28^ observed the inner part of the gingiva exhibits markedly higher ALP activity than the outer area.

The high relative level of Gremlin1 expression suggests that it may play an important role in regulating osteogenesis within the periodontium. Reduction of the non‐specific immunoprecipitation binding was attempted using a number of techniques; however, binding of Gremlin1 to beads was not reduced. The restoration of the inhibitory effect on ALP activity by adding rmGremlin1 to GFCM following treatment using beads suggests Gremlin1 is at least partly responsible for the inhibitory effect of GFCM. Addition of 100 ng/ml rmGremlin1 did not further increase inhibitory activity when added to the untreated GFCM providing further evidence that in the ROS cell assay inhibitory activity is saturated by factor(s), such as Gremlin1, in GFCM alone.

Overall, the data provide compelling evidence that Gremlin1 plays a role in inhibiting osteoblastic differentiation. This is in agreement with existing evidence showing siRNA knockdown of a number of inhibitors including Gremlin1 enhances osteoblastic differentiation.^29^, ^30^, ^31^, ^32^, ^33^ Furthermore, an engineered BMP2 variant that competitively inhibits antagonists has been shown to enhance bone healing in critical size defect model.^34^

Gremlin1 plays an important role in bone development^29^ and has been suggested to be an important marker for identifying osteochondroreticular cells within long bones.^35^ Gremlin1 knockout mice die shortly after birth due to kidney agenesis and also have significant limb bone abnormalities.^36^ Conditional knockout of Gremlin1 targeted to osteoblasts causes a transient increase in trabecular bone volume due to increased osteoblastic activity during growth,^29^ whereas transgenic mice overexpressing Gremlin1 become osteopenic due to decreased bone formation.^37^ Further detailed analysis of the effects on dental development of Gremlin1 overexpression revealed defective enamel and dentine formation with enlarged pulp spaces and alveolar bone resorption at root apices due to inflammation.^38^ The other abnormality of the periodontal tissues observed was that the PDL was less cellular, but no differences in cementum or alveolar bone were noted. This effect of overexpression implies Gremlin1 potentially plays a site‐specific role within the periodontium involving regulation of cell proliferation or differentiation. Overall, this implies BMP inhibitors may have important roles independent of osteogenesis including regulation of cell cycle and potential interaction with other signalling pathways.

## 5 CONCLUSION

Gingival fibroblasts express Gremlin1 inhibiting osteoblastic differentiation mediated by BMP2. Overall, factors secreted by gingival connective tissue inhibit BMP‐induced bone formation, and a range of BMP inhibitors are constitutively expressed in gingival connective tissues. The results suggest these BMP inhibitors, particularly Gremlin1, may play a role in regulating formation and regeneration of bone at the hard‐soft tissue interface.
