# Supplementary material for: Gingival fibroblasts prevent BMP‐mediated osteoblastic differentiation
Source: J Periodontal Res. 2018 Dec 3;54(3):300–9. doi: 10.1111/jre.12631 (PMC6492095; doi:10.1111/jre.12631)
Supplement: Supplementary file 1 [file JRE-54-300-s001.docx]

|  | *Grem1* | *Grem2* | *Nbl1* | *Nog* |
| --- | --- | --- | --- | --- |
| Vector | pBluescript SK- | pBluescript SK- | pT7T3D-PacI | pBluescript SK- |
| Selection gene | Ampicillin | Ampicillin | Ampicillin | Ampicillin |
| Size of insert | 608bp | 555bp | 612bp | 950bp |
| Restriction enzyme to cut for antisense probe | Sal 1 | Eco RI | Not I | Not I |
| Polymerase to generate antisense probe | T3 | T3 | T7 | T7 |
| Restriction enzyme to cut for sense probe | Not I | Not I | Eco RI | Bam HI |
| Polymerase to generate sense probe | T7 | T7 | T3 | T3 |

Table S1. Clones and restriction enzymes used for probe generation for *in situ* hybridization
