# Supplementary material for: Gingival fibroblasts prevent BMP‐mediated osteoblastic differentiation
Source: J Periodontal Res. 2018 Dec 3;54(3):300–9. doi: 10.1111/jre.12631 (PMC6492095; doi:10.1111/jre.12631)
Supplement: Supplementary file 2 [file JRE-54-300-s002.docx]

**Supporting material**

**Supporting Materials and Methods**

**Collagen gel co-culture experiments**

0.5 ml collagen solution (rat-tail collagen type I) (BD Biosciences), sterile 1M NaOH, sterile H_2_0 and α-MEM were applied to 24-well plates and incubated for 30 minutes at 37 ˚C and cells were either added during gelation or subsequent to setting as described later. All cells were resuspended in standard medium before adding to gels.

Two different experimental set-ups were used:

Set-up 1: Simultaneous seeding of osteoblasts and fibroblasts into the gel:

Both osteoblasts and gingival fibroblasts were incorporated together into the collagen gels simultaneously at a density of 5 x 10^5^cells/well calvarial cells, 5x10^5^ or 1x10^6^ gingival fibroblasts. Controls for this experiment consisted of gels seeded only with calvarial osteoblasts at a density of 1 x 10^6^ cells/well. Gels were cultured for 72 hours, and then total ALP levels were measured. Although background levels of gingival fibroblast ALP activity were tested and found to be minimal, they were subsequently corrected in all combined treatments. Briefly, medium was removed leaving the collagen gel containing osteoblasts. Wells were washed with PBS and the contents sonicated by (Soniprep 150) for 20 seconds. 100 μl of sonicates were pipetted into 96-well plate in duplicates. 100 μl of ALP reagent solution per well was added, incubated at 37 °C for 20 minutes in the dark. Plates were then read in a spectrophotometer at 405 nm.

Set-up 2: Sequential seeding of osteoblasts and fibroblasts into the gel:

Osteoblasts were seeded into the setting gel at a density of 5 x 10^5^ cells/well. Fibroblasts were seeded onto the surface of the gel 24 hours later at a density of 5 x 10^5^ or 1x10^6^ cells/well. In control cultures, osteoblasts were seeded instead of fibroblasts after 24 hours, or fibroblasts were seeded during both seeding the gel and after 24 hours. Gels were grown for 72 hours after the second seeding, and then total ALP was measured as described above.

**Osteoblasts grown on fibroblasts matrices**

Fibroblasts or primary calvarial osteoblasts were grown as a monolayer in 96 well-plates at a density of 5 x10^3^ cell/well for 72 hours in standard media. Cells were then killed either by repeating freeze/thawing at -80 °C at least three times or by UV irradiation for 18 hours. Medium was removed and wells were washed with PBS. Osteoblasts were seeded into wells where fibroblasts had previously grown at a density of 5x10^3^ cells/well for 72 hours. Controls consisted of osteoblasts grown without matrices of fibroblasts. Medium was removed and total ALP activity assayed as described previously (12).

**Transwell co-culture**

Osteoblasts were seeded onto the lower level of 24 well plates at a density of 5 x10^4^ cells/well in standard medium. Transwell inserts were added 24 hours later with fibroblasts seeded at a density of 5 x 10^4^ cells/well. Controls consisted of cultures with osteoblasts in both wells and inserts in addition to cultures with wells grown with empty inserts. Cells were grown for 72 hours after seeding the fibroblasts on the inserts then osteoblasts were assayed for total ALP activity as described previously (12).

**Characterisation of conditioned media**

Heat treatment

500μl of each line CM and of control (serum-free media) were immersed in boiling water for 1, 5 and 10 minutes. Samples were then cooled to room temperature and NBCS was added to a final proportion of 10%. ROS cells were seeded in 96 well-plates at 5x10^3^ cells/well. Standard medium was then replaced with heat-treated samples and controls in triplicate. Total ALP activity was assayed as described previously after 72 hours.

Trypsin treatment

CM extracted from three gingival fibroblastic lines were incubated with 1mg/ml of trypsin (Sigma T5266) at 37°C for two hours. Trypsin was then inactivated with 2mg/ml trypsin inhibitor (Sigma T6522) at 37°C for a further two hours. As a control, CM was incubated with 2mg/ml trypsin inhibitor only for two hours. ROS cells were seeded in 96 well-plate at 5x10^3^ cells/well for 24 hours. Standard medium was then replaced with trypsin treated samples and controls supplemented with 10% NBCS in triplicates. Total ALP activity was assayed after 72 hours.

Molecular weight fractionation

Molecular weight filters were used to fractionate gingival CM media from 3 independent primary cell lines using 100 kDa, 50 kDa and 30 kDa filters (Vivaspin, Sartorius UK) by centrifuging at 3000rpm for 15 minutes. ROS cells were seeded in 96 well-plate at 5x10^3^ cells/well. Standard medium was then replaced with concentrate samples, filtrate samples and controls supplemented with 10% NBCS in triplicate. Serum free media fractionated using the same filter sizes acted as controls. Total ALP activity was assayed after 72 hours.

**Migration assays**

A chemotaxis chamber (AP48, Neuro Probe, US) lined with polyvinylpyrolidine-free polycarbonate filters (10μm thickness, pore size 8μm) was used. The filters were pre-coated with type I collagen to promote cell attachment, as described previously (39). Lower wells were filled with either the recombinant mouse (rm)BMP2 or PDGFbb (R&D Systems UK) diluted in conditioned media or controls (serum free media alone). Primary calvarial cells (3x10^5^ cells/ml) were suspended in serum free medium into upper wells. The chamber was assembled and incubated at 37°C for 6 hours. The cells were then fixed by immersing the membrane in ice-cold methanol for 10 minutes. The upper surface where cells were seeded was scraped with the edge of a glass slide six times, and rinsed with PBS to remove any cells left on the upper surface. The membrane was stained with the nuclear stain, DAPI (4’-6-Diamidno-2-phenylindole), cut in two halves and mounted on a glass slides. A fluorescence microscope was used (Nikon Eclipse, TE2000-S) to visualise the cells that had migrated through the membrane. Five fields in each well were taken for cell counting (5 replicates), and three wells were used for each treatment resulting in 15 fields for each treatment.

**Quantitative real-time PCR (qRT-PCR)**

Total RNA was extracted from cultured cells using RNeasy Mini Kit (Qiagen) following the manufacturer’s instruction. RNA purity and concentration was determined using a NanoDrop ND-1000 spectrophotometer (Thermo Scientific). (A_260_/A_280_ 1.8-2 was considered suitable for further analysis). 1µg RNA was reverse transcribed using Moloney murine leukaemia virus (MMLV) reverse transcriptase, nucleotide mix, Oligo dT primers (all Promega). Control reactions omitted reverse transcriptase and were used to confirm the absence of contaminating genomic DNA. qRT-PCR was done on an ABI 7900 7900HT Fast Real-Time PCR System (Applied Biosystems, UK) using ABsolute QPCR ROX Mix (ABgene Ltd., UK) and commercially available pre-designed primer and probes, TaqMan Gene Expression Assays (Applied Biosystems, UK), selected for each gene to be tested. Expression of 8 genes was assessed; Gremlin1 (Grem1) (Rn01509832_m1), Gremlin2 (Grem2) (Rn01412174_m1), Neuroblastoma, suppression of tumorigenicity 1 (Nbl1) (Rn00581091_m1), Noggin (Nog) (Rn01467399_s1), Secreted Frizzled-Related Protein 1 (Sfrp1) (Rn01478473_m1), Bmp2 (Rn01484736_m1), Bmp4 (Rn00432087_m1), and Bmp7 (Rn01528886_m1). Assays for each gene were selected for primers that would detect exons likely to be present in all transcripts. The thermal cycling parameters consisted of an initial hold step of 10mins at 95 °C followed by 40 cycles of 15s at 95 °C and 1 min at 60 °C. The most stable endogenous controls were selected from a panel of 6 using GeNorm software (40) (data not shown). Two control genes were used Eif4a2 and Atp5b which showed the least variation across the samples. The relative quantitative expression of gene of interest, relative to the mean reference gene expression was calculated and this ratio for basal or untreated cells was assigned a value of 1.

Each replicate C_T_ was normalized to the average C_T_ of the controls on a per plate basis by subtracting the average C_T_ of the controls from each replicate to give the
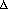
C_T_ which is equivalent to the log_2_ difference between endogenous control and target gene. A raw C_T_ of 35 was set as the limit of detection in this study: individual replicates which gave C_T_ values >35 were considered not detected.

**Immunoprecipitation**

Briefly, 1 µg Gremlin1 antibody (Ab) or controls were incubated with Protein G immunoprecipitation beads (Invitrogen, UK) with rotation overnight at 4 °C to allow the antibody to bind onto the beads. CM were concentrated using Vivaspin 6 ml ultrafiltration devices with 5 kDa molecular weight cut-off (Sartorius, UK) according to manufacturer’s instructions. Excess Ab was removed, beads were washed and 1 ml of concentrated GFCM was added. Mixtures were then incubated with rotation for 3 hrs at 4°C for antigen-antibody binding. Tubes were centrifuged briefly and beads separated from supernatants magnetically (DynaMag-2, Invitrogen). Supernatants were stored at 4 °C for until when needed to for osteoblast proliferation and differentiation assays and western blotting analysis.

**Western blotting analysis**

Protein concentration was determined using the DC Protein Assay (Bio-Rad) according manufacturer’s instructions. Concentrated IP treated media containing liberated proteins (10 µg of total protein) were mixed with Laemmli buffer and subjected to SDS-PAGE. Dual colour marker (Bio-Rad) was loaded as a ladder. Serum-free media was used a negative control. 5 ng/well of recombinant mouse Gremlin1 protein was used as a positive control (R&D Systems). Proteins were transferred onto PVDF membranes and incubated overnight at 4 °C with Gremlin1 primary antibody (Abnova). Secondary antibodies (Dako) (1:5000) conjugated to horseradish peroxidase were then applied for 1 hr at room temperature and proteins visualised via chemiluminescence using ECL Plus detection reagent with Hyperfilm ECL autoradiography film (both GE Healthcare, Bucks, UK).

**Supplemental Figure Legends**

**Supplemental Figure S1. Effect of gingival fibroblasts on ALP activity in collagen matrix culture systems.** Gingival fibroblasts were seeded 24 hrs after seeding of collagen matrices with calvarial osteoblasts. Control: ALP level of 5x10^5^ osteoblasts grown in matrices containing 1x10^6^ osteoblasts. Cal. + Ging.: ALP level of 5x10^5^ osteoblasts grown in matrices with 5 x10^5^ gingival fibroblasts. Cal. + (2xGing.): ALP level of 5x10^5^ osteoblasts grown in matrices with 1x10^6^ gingival fibroblasts. All values normalised to control. ALP activity adjusted for cell number in all experiments. Minimum of 3 replicates per experiment. Data shown as mean+SD. Representative data from three independent experiments are shown. Significant differences shown as * (p<0.05). Significance tested using one-way ANOVA with Bonferroni post-tests.

**Supplemental Figure S2. Normalised ROS cell ALP activity following culture of GFCM treated with trypsin.** Control: standard media. No Rx: neither standard media and GFCM exposed to trypsin treatment. Trypsin and Trypsin inhibitor: incubation of media with trypsin for 2 hours followed by inactivation of residual trypsin with trypsin inhibitor. Trypsin inhibitor only: incubation of media with trypsin inhibitor only. ALP activity adjusted for cell number in all experiments. Minimum of 3 replicates per experiment. Data shown as mean+SD. Representative data from three independent experiments are shown. Significant differences shown as * (p<0.05). Significance tested using one-way ANOVA with Bonferroni post-tests.

**Supplemental Figure S3. Normalised ROS cell ALP activity cultured in GFCM treated with BMP2.** Control: standard media. ALP activity assessed after 72 hours and adjusted for cell number in all experiments. Significant differences shown as *: p< 0.05 to BMP-matched control, †p< 0.05 to baseline control. Significance tested using one-way ANOVA with Bonferroni post-tests
